# O-glycosylation and its role in therapeutic proteins

**DOI:** 10.1042/BSR20220094

**Published:** 2022-10-28

**Authors:** Nicole Thompson, Warren Wakarchuk

**Affiliations:** Department of Biological Sciences, University of Alberta, Edmonton AB, Canada T6G2E9

**Keywords:** glycosylation, O-glycan, protein engineering, therapeutic protein

## Abstract

Protein glycosylation is ubiquitous throughout biology. From bacteria to humans, this post translational modification with sophisticated carbohydrate structures plays a profound role in the interaction of proteins with cells and changes the physiochemical properties of the proteins that carry them. When the glycans are linked to Ser or Thr residues, they are known as O-linked glycans, as the glycosidic linkage is through oxygen. O-glycans are perhaps best known as part of the mucin proteins, however many soluble proteins carry these types of glycans, and that their roles in biology are still being discovered. Many of the soluble proteins that carry O-glycans have a role as therapeutic proteins, and in the 21st century, the application of synthetic biology is starting to be applied to improving these proteins through manipulation of the glycans. This review will explore the role of these O-linked glycans in proteins with pharmaceutical significance, as well as recent advancements in recombinant glycoprotein therapeutics.

## O-glycan biosynthesis

Protein glycosylation in mammals occurs during the biosynthesis of proteins in the endoplasmic reticulum and in the Golgi. The addition of glycans to asparagine is referred to as N-glycosylation [1] and occurs at short sequences known as sequons consisting of a NXT/S motif where X cannot be proline. The addition of glycans to Asn occurs *en bloc* via a lipid carrier and a single glycosyltransferase. This occurs during protein folding and these glycans are then remodeled in the Golgi into hundreds of different structures. In stark contrast with N-glycosylation, the bulk of O-glycosylation occurs in the Golgi after the protein has been folded. Unlike N-glycans, O-glycans are added to proteins one monosaccharide at a time by a myriad of different glycosyltransferases. These O-glycans are initiated by adding N-acetylgalactosamine (GalNAc) [2], fucose, glucose, xylose, or mannose to Ser/Thr residues in a variety of different protein domains [3]. For the purposes of this review, we will restrict the discussion to the glycans initiated by O-linked α-GalNAc.

The human genome encodes 20 distinct isoforms of GalNAc transferases (genes denoted as GALNTX and enzymes denoted as GalNAc-TX) for which the populations of acceptor substrates are largely redundant due to the highly conserved nature of the catalytic domain. These enzymes catalyze the transfer of α-D-GalNAc from UDP-α-D-GalNAc to the Ser/Thr residues. While acceptor sequences are incredibly diverse with over 900 O-glycoproteins identified so far in humans [4,5], sites of O-glycosylation are often characterized by an adjacent Pro residue in position +3 of the modified Ser/Thr [6]. Residue preferences in other adjacent positions for many GalNAc-T isoforms have been well characterized by *in vitro* peptide-based studies [7,8]. Differential expression of these isozymes leads to tissue-specific regulation of the O-glycoproteome, implicating a dynamic role of GalNAc-Ts in the modulation of protein function [5].

**Figure 1:**
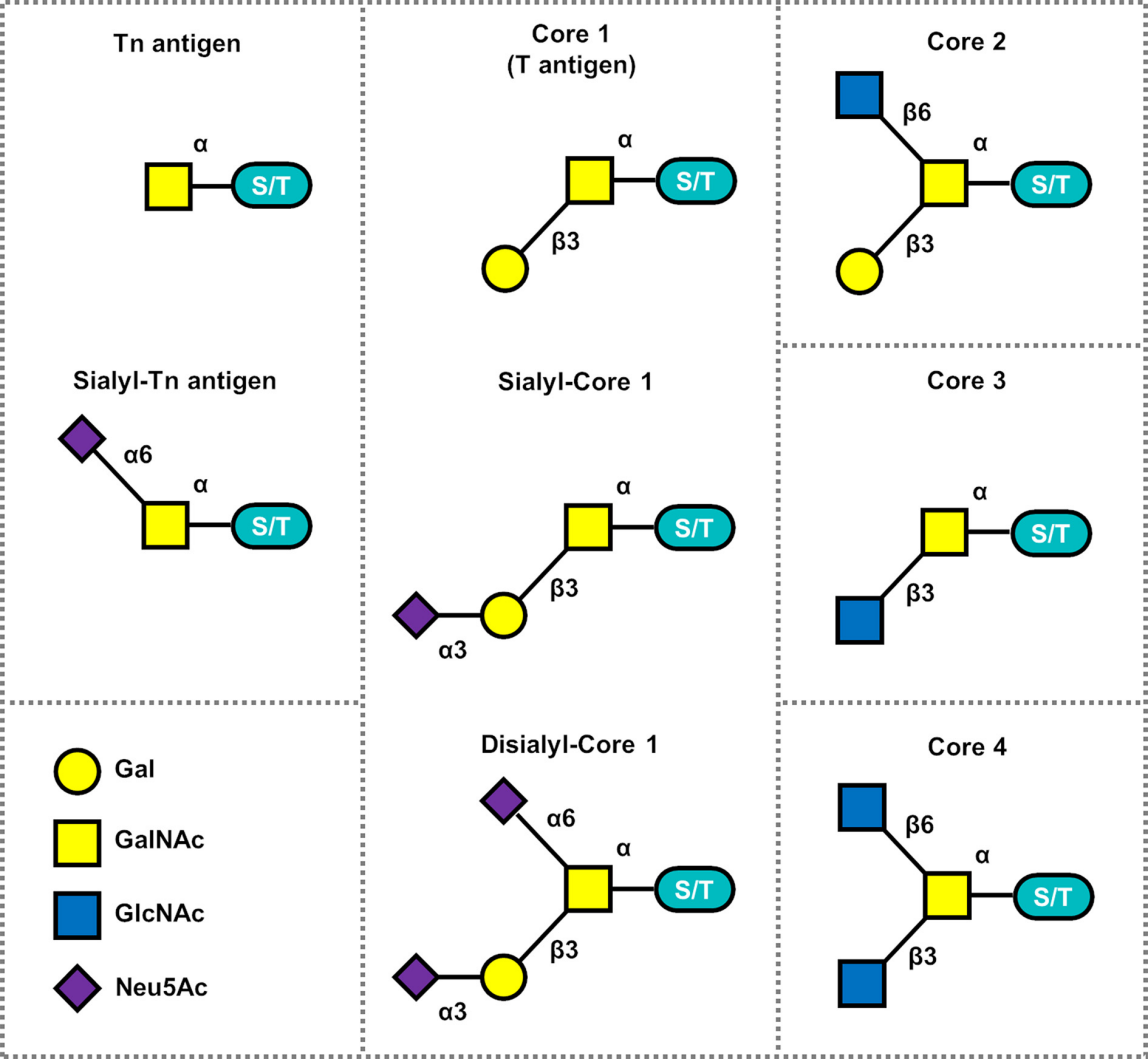
Mucin-type O-glycan core structures and key sialoglycoforms

Further elaboration of O-linked GalNAc produces what are commonly referred to as mucin-type O-glycans owing to the high concentrations of these on mucin proteins. Mucin-like O-glycans are further extended, one monosaccharide at time, into a variety of structures usually referred to by the base structure, or core type. There are four major core structures (Figure 1), and an additional four minor cores structures [2]. The majority of mucin-like O-glycans found on non-mucin proteins are from the core 1 and 2 families. Cores 3 and 4 are mostly restricted to modification of mucins themselves, although there are exceptions to that.

In humans the synthesis of core 1 structure requires two proteins, the glycosyltransferase C1β3GalT (T-synthase), and its specific, endoplasmic reticulum active, folding chaperone COSMC (C1GALTC1) [9,10]. The T-synthase adds Gal from UDP-α-D-galactose in a β1,3 linkage to the α-D-GalNAc on the peptide, this disaccharide is known as the T-antigen (Figure 1, core 1/T antigen). This is further elaborated into core 2 by one of the enzymes C2GnT1-3 – or core 2 synthases, which adds β1,6-linked GlcNAc from UDP-α-D-GlcNAc to the underlying α-GalNAc residue (Figure 1, core 2) [11]. The core 3 structure is elaborated by C3GnT6 [12], which adds β1,3-GlcNAc from UDP-α-D-GlcNAc to the initiating GalNAc. Core 4 structures are made from core 3 precursors by the action of C2GnT2 adding β1,6-GlcNAc from UDP-α-D-GlcNAc to the core 3 base structure [11,13,14]. For the cores 1–3, sialic acid residues can be added by ST6GalNAc1 to the 6-position of the underlying GalNAc from CMP-β-Neu5Ac or in core 1 and 2 by ST3Gal1 to the 3-position of the terminal 3-linked galactose. The GlcNAc residues in core 2/3 structures can be elongated with β1,4 linked galactose and capped by ST3Gal4/6 sialyltransferases.

## Function of O-glycans

The biological roles of O-glycans on proteins continues to be intensely investigated. Elegant work from the Clausen group in Denmark has shown that O-glycans are not restricted to dense mucin-like domains, and that isolated sites of O-GalNAc glycans are indeed very common [4]. Basic functions of these glycans include protection from specific proteases like the case of fibroblast growth factor 23 where a single glycan prevents furin cleavage [15]. The SimpleCell approach described in [4] suggests that there could be hundreds of such sites which then regulate the processing of these proteins. A recent study on peptide hormones suggests that 33% of them carry an O-glycan and that protection from proteolysis and serum half-life are important consequences for these important regulatory molecules [16].

Glycan-mediated protection from proteolysis appears to play a role in coregulating the release of ectodomains of membrane protein receptors by various proteases [17]. One very interesting example is tumor necrosis factor α (TNFα), which is produced as membrane protein that has controlled release from certain cells via the metalloprotease ADAM17 and that O-glycosylation by the GalNAc-T2 isoform plays a role in its release [17,18]. TNFα exhibits toxicity under a variety of autoimmune disease conditions (reviewed in [19]), and so understanding its release could help control these toxic effects. TNFα receptors are also produced in soluble forms [20], which are up-regulated in activated T lymphocytes at sites of chronic inflammation [21], presenting a therapeutic strategy to sequester TNFα and prevent it from doing damage.

There is also a structural role of these glycans on various proteins. For the mucins it adds solubility through water binding to the dense clusters of sialylated O-glycans making them into hydrogels [22]. For non-mucin proteins, the O-glycans frequently appear in proline-rich linker domains where they play a role in the structure and function of the flanking domains, presumably by influencing protein conformation and stability [23]. As will be discussed below, there are some unique and very interesting roles for O-glycans (summarized in Table 1) which are very distinct from the roles played by the perhaps more well studied N-glycans.

**Table 1:** Summary of therapeutic proteins and glycan functions

Protein	Glycan	Functional assignment	Reference
Factor X	Disialylated core 1 on T17/29	Specific engagement with activating proteases FVIIIa/FIXa	[29,30]
Von Willebrand Factor	Disialylated core 1 and some core 2 (∼20%) on T1248/1255/1256/1263	Cluster stabilizes A1 domain, inhibit GPIb binding T1255/1256 glycans involved in plasma maintenance S1486 glycan – platelet binding	[36,39,40]
Interleukin 2	Sialylated core 1 on T3	Decreases aggregation – decreases antigenicity of recombinant protein	[42,48]
Granulocyte colony stimulating factor	Mono/Disialylated core 1on T136	Loop rigidification which prevents unfolding leading to polymeric aggregates which are inactive. Possible protection from proteolysis	[53,54]
Granulocyte-macrophage colony stimulating factor	Sialylated core 1 on S5/7/9 T10. There is variability on the number of sites that are occupied in a given preparation	Not known. Possible protection from antibody formation to the recombinant protein	[60,64]
Interferon α	Disialylated core 1 on T106	Not known. Perhaps decreasing aggregation/antigenicity?	[66,96]
Erythropoietin	Disialylated core 1 on T126	Not known.	[70–72]
Etanercept	Mono- and disialylated core 1, sialyl-Tn, and sialylated extended core 3 – T8/184/200/245 and S199	Increase in serum half-life, and increased TNFα affinity	[83,84,86]
C-terminal peptide of chorionic gonadotropin β – as a fusion with human growth hormone, FVIIa, FIX, IFNb1a, IFNa2b	Sialylated core 1	Increase in serum half-life	[89–93]
Tagged interferon α 2b	Unknown – sialylated core 1?	Increase in serum half-life	[98]

### Coagulation factors: Factor VIII, IX and X

Hereditary bleeding disorders, hemophilia A and B, result from mutations in proteins used for the coagulation cascade, broadly known as coagulation factors. The proteins in this cascade are made as inactive zymogens carrying many post-translational modifications [24], which are then activated through specific proteolysis events. Hemophilia is treated by supplying exogenous blood factors to the patients through infusions. Factors VIII and IX are used in the treatment of hemophilia A and B, respectively [25], and are produced from pooled human serum or as recombinant proteins in tissue culture which either preserves their native glycosylation, or in the case of tissue culture derived cells, presents under-glycosylated and other possibly antigenic glycans [26]. Under-glycosylation and non-human glycotypes may lead to eventual inhibition of the exogenously added therapeutic protein [27]. Both proteins are involved in activating Factor X (FX) to Factor Xa (FXa), which is then the major generator of thrombin needed to push the clotting process forward. The glycosylation of these proteins has been extensively studied, however mostly in the context of the N-glycans. The O-glycome has however been investigated for many plasma-derived proteins including coagulation factors [28], as well as recombinantly produced forms [26].

**Figure 2:**
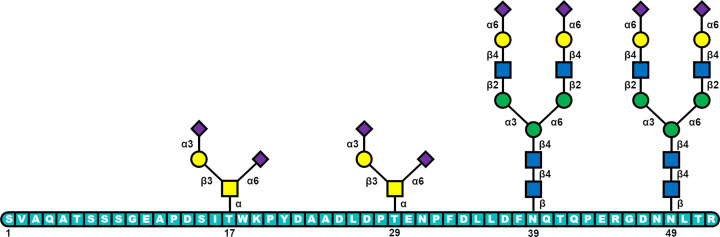
Schematic of the Factor X activation peptide The most common glycans identified are shown at positions T17/29 and N39/49.

Early work on the role of glycosylation was contradictory, but more recent detailed studies have revealed an intriguing role for the O-glycans on the FX activation peptide (Figure 2). Studies on FX have shown the presence of O-glycans at positions T17 and T29 play a positive role in interaction with FVIIa and FIXa in the activation of the FX zymogen when it is in contact with the intrinsic co-factor FVIIIa [29]. In the present study, site-directed mutagenesis of T17/T29 and N49 revealed that the presence of disialyl-core 1 O-glycans structures was required for a positive, specific interaction with the activating intrinsic FVIIIa protease. This was shown through measuring the activation rates without the O-glycans and observing they were significantly impaired for the intrinsic FVIIIa. The N-glycan at N49 on the activation peptide seems to function to prevent the unregulated action of free (extrinsic) FVIIa and FIXa to activate FX but has no effect on the intrinsic co-factor dependent proteolytic reaction. A very recent paper from a group at Novo Nordisk in Denmark [30] reinvestigated some of these findings through a detailed site-directed mutagenesis study. They ignored the N-glycan at N49 and instead looked at the N-glycan at N39, which was not considered in the previous paper. In terms of the O-glycans, they also concluded the O-glycan at T17 was required for specificity of the interaction with FIXa for the FX zymogen/FVIIIa complex. Contrary to the Yang et al. paper, they found no measurable effect of the T29A mutant. However, there were some differences in the sequence of the activation peptide in the Yang et al. study which may have contributed to that difference.

While both studies concluded a similar role for the T17 O-glycan, there was no determination of what the glycans were on the mutant proteins. This could be important particularly for the O-glycans at T17/29 as the presence or absence of the initiating GalNAc could change the glycan at the other position [31]. While this needs to be further investigated, the fact remains that for the FX activation O-glycans play a regulatory role.

#### Von Willebrand factor

Another protein involved in bleeding disorders is von Willebrand factor (VWF). Von Willebrand disease (VWD) is the most common inherited bleeding disorder, affecting approximately 1% of the population in the United States (https://www.cdc.gov/ncbddd/vwd/data.html). Treatment is through a variety of products, but recombinant VWF is currently the best option [32]. The VWF protein is involved in hemostasis and binding of its A1 domain to platelets occurs through the platelet receptor, Glycoprotein Ib (GPIb). VWF also interacts with and stabilizes FVIII in the blood [33]. VWF is a very large glycoprotein, which undergoes concatemer formation that influences platelet binding. Under low shear conditions such as normal circulation VWF does not bind platelets, but when exposed to elevated shear, tension causes the protein to elongate and opens the structure to allow the A1 domain to bind platelets [34,35] and then go on to form a platelet plug. What is fascinating is that the presence of O-glycans on the linkers of domain A1 to its adjacent domains is critical for this activation [36].

**Figure 3:**
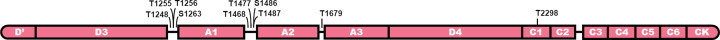
Schematic of von Willebrand factor domains and O-glycosylation sites The cluster of sites between the D3/A1/A2 domains are labelled with residue numbers.

VWF has 10 O-glycan sites, occupied mainly with disialylated core 1 (∼78%), but with some core 2 (∼21%), and approximately 1% ABO blood group containing structures on the core 2 branch [37]. These glycans occur in two clusters on linkers flanking the A1 domain (Figure 3) and have been shown to have a negative regulatory effect on A1 domain affinity for GPIb [38].

In a series of *in vivo* experiments [39], the T1255 and T1256 O-glycans were shown to play a role in plasma maintenance of VWF, while the S1486 O-glycan plays a role in platelet binding. In a series of biophysical experiments to quantitate GPIb binding to the A1 domain [36], the N-terminal cluster of four O-glycans were shown to strongly inhibit binding of A1 to the platelet GPIb protein under normal conditions, indicating allosteric regulation of GPIb affinity in addition to the known physiological induction by tensile force. The O-glycans also stabilize the A1 domain, which decreases GPIb protein binding. Energy input when VWF experiences higher shear force causes a change in the linker conformation which opens the binding site for GPIb, and the negative charge density of sialic acids (8 in total) at these four sites may also have a repellant electrostatic effect on GPIb binding. In addition to the electrostatic interaction, a recent paper highlights that sialylation of the O-glycans also contributes to plasma half-life by protecting the protein from clearance through the macrophage galactose lectin [40]. This represents an interesting case where N- and O-glycans cooperate to increase plasma half-life, as the sialylated N-glycans protect the protein from liver clearance through the Ashwell–Morell receptor.

These observations are consistent with the widely held idea that mucin type O-glycans add stability to the protein domain they are associated with and also suggest that the density often associated with O-glycan modification may have an electrostatic repulsive force to prevent binding. This contrasts with the role that O-glycans play in the activation of FX, where they are involved in a positive manner by enhancing specificity of binding. The cooperative interaction between N- and O-glycans to extend plasma half-life for VWF is something that probably plays a role on other non-mucin proteins with both types of glycosylation, and it will be interesting to see which proteins those are and how general this phenomenon is.

### Cytokines

**Figure 4:**
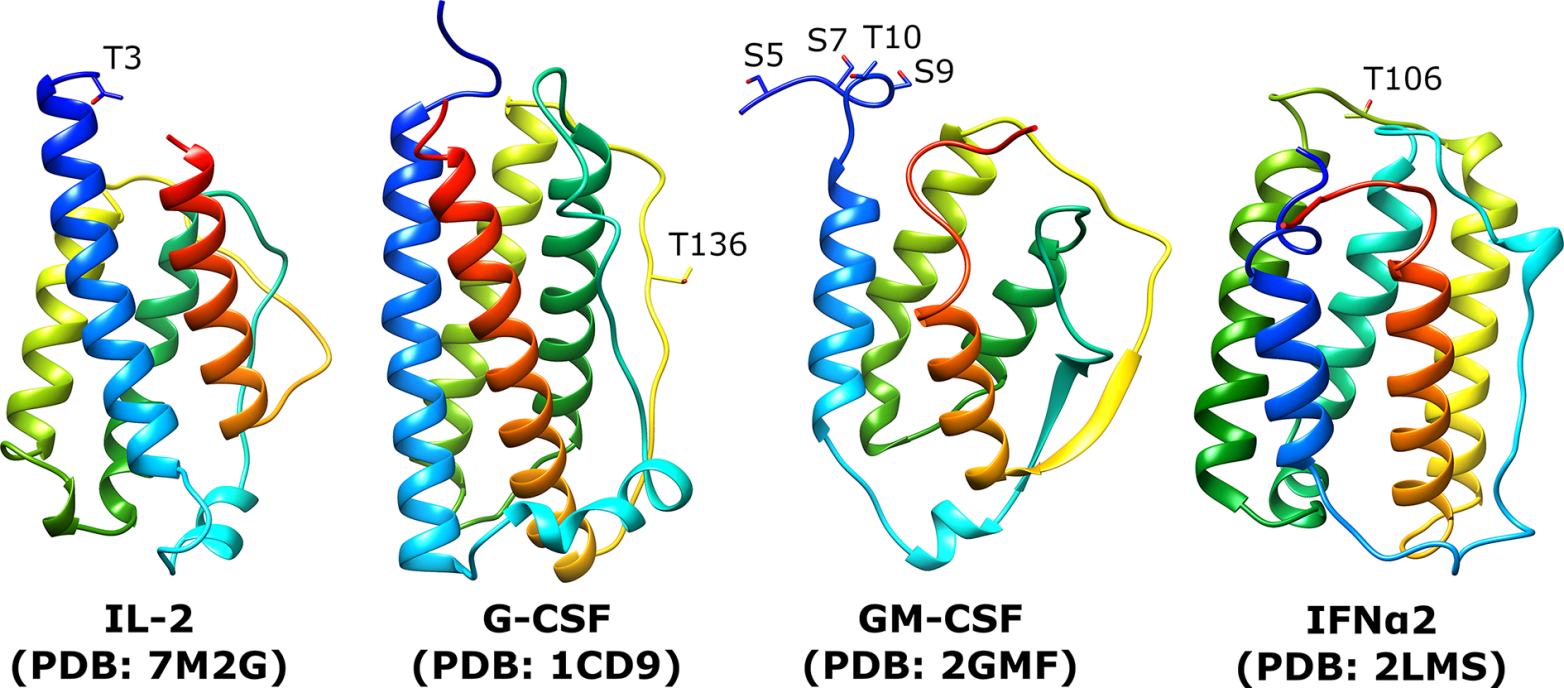
Structures of human glycoprotein therapeutics IFNα2 (PDB: 2LMS), IL-2 (PDB: 7M2G), G-CSF (PDB: 1CD9) and GM-CSF (PDB: 2GMF) Chains are colored blue to red from N- to C-terminus. Sites of O-glycosylation are labeled by amino acid position of the mature peptide.

Cytokines are important signalling proteins that occur as gene families giving rise to many of these proteins having some overlapping biological activity [41]. The interleukins, interferons and colony stimulating factors share a similar protein fold (Figure 4). Given their potent biological activity, several of them are in clinical use or development. From a glycoprotein perspective they are somewhat enigmatic as only certain members of the families are glycosylated, and as we will discuss, they often have only one O-glycan.

#### Interleukin-2

Interleukin-2 (IL-2) is a cytokine that plays an important role in the induction of T-cell proliferation. It was originally described almost 40 years ago as being O-glycosylated on an N-terminal residue (T3 of the mature peptide), and this single site of glycosylation carries a sialylated core 1 glycan when produced by Jurkat cells [42]. The protein has been studied as a therapeutic for many years but has suffered from short serum half-life and toxicity issues depending on the dose [43]. In the cited review from 2019, there is no mention of the glycosylated form of the protein, and in fact the recombinant form of IL-2 has mainly been produced in *Escherichia coli* for decades and so has not been natively glycosylated since the early tests as a therapeutic [44]. It was concluded that the glycosylation did not play a role in the functioning of IL-2. The use of IL-2 has presented challenges right from the earliest uses, and these are related to the dosage required and the unmodified protein’s short half-life [45]. Problems with toxicity have limited the use of IL-2 to metastatic melanoma and kidney cancer [45]. Several modified versions of IL-2, including PEGylated and Fc fusions have also been produced in attempts to improve serum half-life and dosing issues [43].

So, if the N-terminal O-glycan is not required for activity, what is it for? When recombinant IL-2 became available for treatment, patients were seen to mount an immune response to the *E. coli* produced protein, but it was noted that the native, glycosylated form did not elicit an antibody response. These early and extensive data are reviewed in [46]. Many therapeutic proteins lead to antibody responses against them [47], and IL-2 was one of the first proteins to be used clinically and for which the antibody response was subsequently linked to the lack of the N-terminal glycan at T3. The idea that has been suggested for the protective effect is that the O-glycan decreases aggregation of the protein, and that the aggregates are the immunogenic form [48].

Narrowing in on the roles of specific O-glycan structures, a synthetic protein approach has recently been reported where homogeneous glycoforms of IL-2 were prepared and the biological consequences measured [49]. Using semi-synthesis via a serine ligation strategy, these researchers assembled four variants with simple GalNAc, core 1, core 1 with PEG, or sialylated core 1. The assessment of biological activity was not extensive but the suggestion from this work, was that the sialylated glycoform had the weakest biological activity, only 57% when activating T-cell proliferation. This is a very preliminary result, but it points to the kind of study that is needed to understand how native glycoforms affect the biological activity of these proteins.

#### Colony stimulating factors

Colony stimulating factors are a group of cytokines which stimulate the production of various myeloid cell linages. Granulocyte colony stimulating factor (G-CSF) is an important therapeutic used to stimulate cells of the neutrophil lineage to maturation, and thus increase the level of neutrophils. It is an effective and widely used therapy for neutropenia. This cytokine was identified as a hydrophobic glycoprotein in 1985 [50], and as with IL-2 discussed above, the single O-glycan at T136 was shown not to be required for biological activity, but several reports suggested the glycosylated form was more potent [51]. However, a meta-analysis of clinical usage published shortly afterward concluded there was no difference in biological potency between the two forms [52].

Shortly after its debut as a recombinant therapeutic, the role of the glycan was investigated on two different fronts. The first was a study showing that un-glycosylated G-CSF was prone to aggregation and an accompanied loss of activity compared to the single disialylated core 1 form of the protein [53]. Unlike what was cited above for IL-2, stimulating antibodies to rG-CSF does not appear to have been cited as a mechanism of loss of potency. One suggestion for a function of the glycan has been that the glycan confers structural stability to the protein, which was suggested through NMR experiments [54], the idea being that the glycan rigidifies the loop that carries it. This rigidification then could help with events that would lead to unfolding.

A second function for the single O-glycan on G-CSF that has been investigated is protection of the protein from neutrophil elastase [55]. While the present study showed that glycosylated G-CSF was partially protected from human neutrophil elastase, these were *in vitro* assays. In the same paper, when human serum was incubated with an elastase inhibitor, the non-glycosylated G-CSF was still inactivated, so elastase is not the only factor involved in the serum induced inactivation of G-CSF, and we will need more detailed investigation to unravel serum inactivation of these proteins.

Granulocyte-macrophage colony stimulating factor (GM-CSF) acts on bone marrow cells to generate colonies of granulocytes, macrophages, or both. GM-CSF is used in cancer therapy to augment the immune response to tumour cells [56] but also has efficacy in increasing the immune response to vaccines (those studies are reviewed in [57,58]). GM-CSF was first purified from the conditioned media of mouse lung cells where it was noted to be a glycoprotein, but the glycans were not characterized [59]. Unlike the cytokines discussed above, later investigation of the glycans through site-directed mutagenesis revealed that GM-CSF contains both N- and O-linked glycans, with two N-linked sites and four O-linked sites [60]. The roles of these glycans were investigated *in vivo*, where the plasma half-life was linked to the N-glycans as for other N-glycan containing proteins [61], but the O-glycan has not really been investigated other than to say it is not needed for biological activity [60–62].

There was an early report that perhaps the O-glycans masked an epitope for antibody production [63], but the data were certainly not conclusive. In the present study, 4/16 patients given recombinant GM-CSF from yeast (partial N-glycans and no O-glycans) or *E. coli* (no glycans) developed antibodies to the recombinant protein, though no evidence of neutralizing activity was found. The thought was that the bare N-terminal region was where the antibodies bound, and that these antibodies facilitate clearance from the blood. It is interesting that for this protein, several studies had been done without knowing what the O-glycan structures were. The O-glycan identity remained unresolved until a 2004 paper [64] showed they were monosialylated core 1 structures. In a more recent study, the appearance of antibodies to GM-CSF in Crohn’s disease patients was shown to be correlated with aberrant N-glycans. However, again the O-glycans were ignored completely, so it is not clear if they also are modified in these patients [65].

So, once again we are left with an incomplete picture of what the role of those O-glycans are! Is it a combination of protein stability, and anti-aggregation? It appears that there are still any unanswered questions which need to be addressed, and which could be addressed with modern molecular biology approaches, including homogeneous glycoform synthesis, which has been demonstrated for the N-linked sites on GM-CSF using *in vitro* peptide ligation [62].

#### Interferon α

Another member of the group that needs to be mentioned is interferon α 2b (IFNα2b). A paper published in 1991 [66] established that IFNα2b carried a single O-glycan on T106. As this protein was already being used a therapeutic but as a recombinant protein produced in *E. coli*, the glycan was concluded to be unimportant for its biological activity. IFNα2a/b have been used clinically very successfully, mostly as material derived from *E. coli* expression. The native material and *E. coli* produced proteins have short plasma half-lives, and so as with other cytokines PEG modified material has been used clinically and is reviewed in [67]. Unlike the other cytokines mentioned in this section, there has been very little investigation into what role this single glycan plays in the natural protein. However, as we shall see in the next section, glycosylation has been looked at for engineered variants of IFNα.

#### Erythropoietin (EPO)

A very well-known therapeutic protein from the cytokine superfamily that has been intensively studied from the perspective of its glycans is EPO. This cytokine is produced by the liver and stimulates red blood cell production, it was first shown to be a glycoprotein in 1959 [68]. This particular protein also carries a single disialylated core 1 glycan at T126, but this has been overshadowed by the 3 natural N-glycans, or the 5 N-glycans on the engineered version, Darbepoetin alfa [69]. Studies aimed at understanding the role of glycans quickly dismissed the O-glycan as un-important after removing it genetically and seeing no change in secretion or *in vivo* activity [70,71]. An extensive examination of the glycans on the recombinant forms of EPO has shown that sialic acid acetylation, particularly on the O-glycan is found in the engineered form Darbepoetin alfa [72]. O-acetylated sialic acids can be difficult to study as they are not very stable – perhaps this transient modification is important? As we saw with interferon α, there has been no function ascribed to this tetra-saccharide, so further studies are needed to find the role for this glycan.

## Glycoengineering strategies

Recombinant protein production became a reality for therapeutic proteins nearly 50 years ago. In that time, the clinical application of these proteins has encountered significant problems with short serum half-life or antibody responses as noted above. Currently, a widely adopted approach for extending the serum half-life of drugs is through modification with large polymers of polyethylene glycol (PEG). However, there is emerging evidence that the widespread use of PEG in both medicinal and consumer products has led to a prevalence of pre-existing IgG and IgM antibodies against PEG in the general population [73]. These anti-PEG antibodies have been linked to first-exposure allergic reactions [74], inhibition of drug activity [75] and accelerated blood clearance [76]. Given the potential implications of anti-PEG immunity, alternative strategies are sorely needed as protein engineering shifts to the forefront as the driving force for improved therapeutics. Glycosylation continues to be a critical factor in therapeutic proteins [77,78], and along with protein engineering, glycan engineering is also being applied to these crucial therapeutics [79–82].

### Glycosylated fusion partners of therapeutic proteins

A rapidly growing class of approved biopharmaceuticals make use of the IgG1 Fc region as a fusion partner to extend serum half-life. In some cases, this has led to a concerted benefit where the fusion protein has a greater number of occupied O-glycosylation sites than what is found on the native proteins individually. Perhaps the best example of this is etanercept, a dimeric Fc fusion of human tumor necrosis factor receptor p75 (TNFR2) used as a TNFα sequestrant in the treatment of autoimmune diseases, which contains predominantly sialylated core 1 O-glycans [83]. The sites of O-glycosylation are mostly located in the linker region and are more numerous than what has been reported for the native TNFR2 extracellular domain. There is also one site on the Fc hinge portion of this linker that is not normally found on IgG1 [84]. Sialylation of these glycans enhances serum half-life by masking terminal galactose residues, preventing clearance via asialoglycoprotein receptors in the liver [85]. More recently, a variant produced from CHO cells overexpressing ST6GalNAc1 to generate a mixture of disialyl-core 1 and sialyl-Tn as the major glycoforms increased TNFα affinity as well as potency [86]. Interestingly, the same study observed that Cosmc-knockout CHO cells overexpressing either ST6GalNAc1 or C3GnT, to generate the truncated Tn/Sialyl-Tn or sialylated core 3 tetrasaccharide structures respectively, also influenced TNFα affinity but not potency.

Unexpected glycosylation of the IgG1 hinge region has also been observed on atacicept, with a second O-glycan in addition to the one identified on etanercept [87]. This protein is a dimeric Fc fusion of the transmembrane activator, calcium-modulator, and cyclophilin-ligand-interactor (TACI) receptor, which is another member of the TNF receptor superfamily. Rounding off this trend is abatacept, a dimeric Fc-fusion of the cytotoxic T-lymphocyte-associated antigen 4 (CTLA-4), with a total of four O-glycans identified in the hinge region [88].

Alternative fusion partners to improve drug efficacy often come in the form of known glycosylated domains. An early example of this was the fusion of follitropin β (FSHβ) with the natively O-glycosylated C-terminal peptide (CTP) of chorionic gonadotropin β (CGβ) [89]. Having two tandem CTP repeats conferred a significant benefit to both potency and *in vivo* half-life. The CGβ CTP has since found similar success as a fusion partner for human growth hormone (hGH) where the increased half-life compared with wild-type hGH was enough to compensate for its decreased *in vitro* potency, allowing for less frequent dosing [90]. This long-acting hGH, somatrogon, is produced in CHO cells and contains three total CTP repeats flanking the hGH sequence (one N-terminal and two C-termini). It has since completed phase 3 clinical trials, with weekly administration showing similar efficacy to daily administration of somatropin, a recombinant hGH produced in *E. coli* [91]. Similar CTP fusions have also been investigated for FVIIa, FIX and IFNβ1a [92], as well as IFNα2b [93].

More recently, this approach has gained broader traction with cytokines to overcome short serum half-life. One such study explored the idea of a tagged IL-2 using the densely O-glycosylated hinge region of natural cytotoxicity triggering receptor 2 (NCTR2) [94]. This tagged protein is roughly double the size of native IL-2 and certainly has an improved serum half-life, but its biological activity appears to have been altered. The anticancer effect of the new fusion protein is less than the native protein, and it appears to function as a mediator of anti-inflammatory activity which might have some value in treating autoimmune diseases. This points to the caveat that for a protein with such potent biological activity, such a highly modified protein might be undesirable. A disappointing feature of this paper was that the actual glycans present on their recombinant protein were not determined, and so meaningful conclusions about the role of specific O-glycans in the function of the engineered fusion could not be made.

One last example from the cytokine group is a sequence tagged version of IFNα2b [82,95]. IFNα2b is widely used as an antiviral therapy, but it does suffer from short plasma half-life and as mentioned PEGylation might not be the best strategy for increasing plasma half-life. There are also several reports of antibodies being generated during treatment with the recombinant form of IFNα2 [96]. The study from Sales et al. follows up on work they did to make a hyperglycosylated version of IFNα2b with 4 N-linked glycans which while having a great plasma half-life was possibly increased in antigenicity [97,98]. Their new strategy for an engineered IFNα2b is related the sequence tagging approach used above for hGH, and IL-2, but in this case using the N-terminal sequence of GM-CSF. This protein contains either the first 14 amino acids of GM-CSF or a modified version of that sequence ‘APARSPSPTPTPTPT’. This gives seven potential sites of glycosylation, including the natural T106 and mass shifts on SDS-PAGE show an increase in mass – but again no accurate determination of the protein mass or identification of the glycans attached to the sequence tag make it difficult to interpret which glycans are added – or how heterogenous this may be. One potentially interesting finding is that while antiviral activity is similar for the WT IFN and the two variants, the antiproliferative activity was decreased for the modified sequence tag. Again, it would have been informative to know if there were glycoform differences between the two tagged versions that lead to the decrease in anti-proliferative activity.

### Site specific pathogen glycoconjugate vaccine candidates

Pathogenic bacteria normally make cell surface glycans which have long been used in vaccine production as conjugates to carrier proteins as exemplified with multi-valent Streptococcal vaccines [99]. Synthetic biology and recombineering have made second generation glycoconjugates as O-linked glycan polymers an important area for development. This area has recently been reviewed [100]; however as we are going to cover engineering bacteria to make O-glycans on other therapeutic proteins, it is appropriate to mention here the basic system for these novel O-linked glycans on protein carriers.

Bacteria use an oligosaccharyltransferase (OT) to link glycan polymers formed on undecaprenyl carrier lipids to lipid anchors and proteins on the outer surface of the bacterial cell. Two types of OT were originally described, PilO, and PglL, which are found in a variety of pathogens and have specific requirements for the size and kind of terminal monosaccharide that can be transferred to make an O-glycan linkage [101]. This technology has been applied to a variety of protein conjugates as vaccine candidates [102], but in 2019 a well-developed platform was described using an OT variant known as PglS which can use glucose as the linking sugar [103]. This was used to make three Streptococcal antigen conjugates and demonstrates the utility of the system for site specific conjugates on suitable carrier proteins. These observations demonstrate that synthetic biology is changing how conjugate vaccines are produced.

### Therapeutic proteins and glycosylation pathway engineering for human glycans

Efforts to introduce human mucin-type O-glycosylation into proteins produced in *E. coli* have also made use of O-OTs, such as PglO from *Neisseria gonorrhoeae* and PglL from *Neisseria meningitidis*, to transfer preassembled glycan structures *en bloc* from a lipid carrier [104]. This approach also employed significant reengineering of metabolic pathways within the *E. coli* host to avoid unwanted undecaprenyl lipid-GlcNAc precursors. In the present study, the authors showed that the core 1 could be quite effectively transferred to peptide fragments of human mucins (up to 41 amino acids) on a maltose binding protein carrier protein. Attempts to get sialylated core 1 structures were successful with the enzymes chosen for the present study; however, most of the glycan modification on these fusions was not sialylated. This study does present some limitations as an authentic therapeutic protein was not used as a target, and these enzymes depend on the target protein getting to the periplasmic space. Finally, the spectrum of bacterial enzymes to build authentic human O-glycans on the undecaprenyl lipid carrier is limited and will require further protein engineering before that can be fully realized.

Integrating glycoengineering approaches into bacterial expression platforms is an attractive strategy for cost-effective production of extended half-life biologics. Given the ability of some bacteria to mimic host glycans as a strategy to evade host immune response, the diversity of bacterial glycosyltransferase activity offers a convenient pool of bacterially endogenous enzymes with which to intentionally engineer strains to produce human-like glycans. Work from the author’s laboratory has been addressing this question. We have seen the likelihood of success of this approach depends on enzymes that have been characterized as promiscuous for unnatural substrates. One such example is the expression of core 1 glycoproteins in *E. coli* without the requirement of the Cosmc-dependent T-synthase by leveraging the *Campylobacter jejuni* CgtB to galactosylate the Tn antigen to make core 1 [105]. In combination with genomic integration of the *neuCAB* operon for the sialic acid donor substrate (CMP-Neu5Ac) biosynthesis from *Neisseria meningitidis*, this plasmid-based expression system was expanded to produce authentic sialylated forms of recombinant therapeutics [106]. This was the first demonstration of using isoform specific O-glycosylation prediction (ISOGlyP) [107] to guide site-directed mutagenesis and was successful in improving *in vivo* glycosylation efficiency of IFNα2b as well as introducing a novel O-glycosylation site in human growth hormone.

Closer examination of the underlying relationship between ISOGlyP score and enzyme activity of the human GalNAc-T2 revealed a positive correlation with V_max_ and overall catalytic efficiency – an effect that is also seen in orthologues from *Drosophila melanogaster* and *Caenorhabditis elegans* [108]. While there are certainly complicating factors introduced by the secondary and tertiary structure of protein substrates that ISOGlyP cannot account for, it is proving nonetheless to be an incredibly valuable tool in glycoprotein engineering.

What is also clear from this work, is that certain mammalian enzymes which naturally use protein substrates can be produced functionally in the cytoplasm of *E coli*. In the study above, three mammalian glycosyltransferases each with 3 disulfides are expressed well enough to produce disialyl-core 1 glycans at the specified location on a properly folded human proteins (which also have disulfide bonds). The next steps for this work are to extend the number of enzymes that can be added to hopefully produce other common core types that would be of benefit for further research into what these O-glycans are contributing to the protein’s function.

## Conclusions

Compared to N-linked glycans, O-linked glycans are very understudied. In the context of normal biology, many functions have been ascribed to O-glycans [4,5,109,110]. This review has pointed out that in some cases a specific role for an O-glycan has been investigated e.g. FX activation, but in many other cases careful study of the role of the O-glycan has been neglected, e.g., cytokines and colony stimulating factors. Some research has been started to make molecules with defined glycans, but it has been limited. As we saw with proteins like etanercept, changes in the O-glycan to rare glycoforms like the sialyl-Tn and the sialylated core 3 tetrasaccharide can influence the ligand binding properties of this therapeutic. The advent of synthetic biology has provided us with the tools to address some of these questions potentially with single glycoform versions of proteins.

The guiding of O-glycan design to effect function is a goal of glycoengineering; however, we are not yet able to predict what structures are the ‘best’ for a certain protein. It will require us to make use of a few robust bioassays, and potentially dozens of constructs with addition/deletion of enzymes to make a library of protein glycoforms. We have the tools for improved, tailored therapeutics with O-glycans and this is an area of research of great potential. Either in tissue culture or possibly in microbial expression systems a variety of approaches are now possible to advance our understanding and application of O-glycosylation for therapeutic proteins.

## Abbreviations

CGβ: chorionic gonadotropin β
CTLA-4: cytotoxic T-lymphocyte-associated antigen 4
CTP: C-terminal peptide
EPO: erythropoietin
FSHβ: follitropin β
ISOGlyP: isoform specific O-glycosylation prediction
NCTR2: natural cytotoxicity triggering receptor 2
OT: oligosaccharyltransferase
PEG: polyethylene glycol
TNFα: tumor necrosis factor α
VWD: von Willebrand disease
VWF: von Willebrand factor
